# The Meiotic Recombination Checkpoint Suppresses NHK-1 Kinase to Prevent Reorganisation of the Oocyte Nucleus in *Drosophila*

**DOI:** 10.1371/journal.pgen.1001179

**Published:** 2010-10-28

**Authors:** Oscar M. Lancaster, Manuel Breuer, C. Fiona Cullen, Takashi Ito, Hiroyuki Ohkura

**Affiliations:** 1 The Wellcome Trust Centre for Cell Biology, School of Biological Sciences, University of Edinburgh, Edinburgh, United Kingdom; 2 Department of Biochemistry, Nagasaki University School of Medicine, Nagasaki, Japan; Stowers Institute for Medical Research, United States of America

## Abstract

The meiotic recombination checkpoint is a signalling pathway that blocks meiotic progression when the repair of DNA breaks formed during recombination is delayed. In comparison to the signalling pathway itself, however, the molecular targets of the checkpoint that control meiotic progression are not well understood in metazoans. In *Drosophila*, activation of the meiotic checkpoint is known to prevent formation of the karyosome, a meiosis-specific organisation of chromosomes, but the molecular pathway by which this occurs remains to be identified. Here we show that the conserved kinase NHK-1 (*Drosophila* Vrk-1) is a crucial meiotic regulator controlled by the meiotic checkpoint. An *nhk-1* mutation, whilst resulting in karyosome defects, does so independent of meiotic checkpoint activation. Rather, we find unrepaired DNA breaks formed during recombination suppress NHK-1 activity (inferred from the phosphorylation level of one of its substrates) through the meiotic checkpoint. Additionally DNA breaks induced by X-rays in cultured cells also suppress NHK-1 kinase activity. Unrepaired DNA breaks in oocytes also delay other NHK-1 dependent nuclear events, such as synaptonemal complex disassembly and condensin loading onto chromosomes. Therefore we propose that NHK-1 is a crucial regulator of meiosis and that the meiotic checkpoint suppresses NHK-1 activity to prevent oocyte nuclear reorganisation until DNA breaks are repaired.

## Author Summary

Meiosis is a specialised form of cell division that produces haploid gametes from diploid cells. Failures or errors in meiosis can lead to infertility, miscarriages, or birth defects. In meiosis, chromosomes first swap genetic information during recombination and then undergo two rounds of segregation. Temporal separation of these distinct meiotic events is essential for successful meiosis. To ensure this correct temporal order, the meiotic recombination checkpoint blocks meiotic progression when recombination is not completed. Adding to our understanding of this process, we here report that the conserved *Drosophila* protein kinase NHK-1 is a crucial regulator of meiosis that is controlled by the meiotic recombination checkpoint. The meiotic recombination checkpoint suppresses the activity of NHK-1 to block transitional remodelling of meiotic chromosomes in the oocyte nucleus until recombination is completed.

## Introduction

Meiosis is a specialised form of cell division that differs from mitosis in many respects, particularly during the exchange of genetic information between homologous chromosomes in recombination. In early meiotic prophase, DNA double-strand breaks (DSBs) are introduced into meiotic chromosomes by the conserved enzyme Spo11 to initiate recombination [1]–[4]. An elaborate structure, the synaptonemal complex, then forms between homologous chromosomes stabilising their pairing and recombination [5]. Once recombination is complete and DSBs have been repaired, the synaptonemal complex is disassembled. As these events are meiosis-specific, molecular mechanisms of meiotic prophase progression need to be established beyond our understanding of mitotic cell cycle control.

Eukaryotes have a surveillance-signalling system, the so-called meiotic recombination checkpoint (hereafter referred to as the meiotic checkpoint), which prevents meiotic progression until DSBs generated during recombination are repaired [6]–[8]. Many advances have been made recently in determining the mechanisms involved in the detection of and signalling downstream from DSBs [9]. In contrast, little is known about how the checkpoint signal blocks meiotic progression, except in yeast. In yeast, the Cdc28 (Cdk1)-Cyclin complex is suppressed in various ways by the meiotic checkpoint to delay or block meiotic division [10]–[12].

In *Drosophila*, the meiotic checkpoint was first revealed by the study of a class of mutants collectively called *spindle* (*spn*) mutants. These *spn* mutants were originally identified based on their abnormal dorsal-ventral oocyte polarity [13]–[16]. They also share abnormalities in a meiosis-specific organisation of chromosomes called the karyosome [14], [17], [16].

The meiotic checkpoint pathway is activated in *spn* mutants by persistent DSBs caused either by defects in DNA repair during recombination [18], [19] or in processing of repeat-associated siRNA that suppress germline retrotransposition [20]–[22]. Signalling downstream of DSBs in the meiotic checkpoint requires the successive activation of two conserved kinases, Mei-41 (an ATM/ATR homologue) and Mnk/Chk2 [17], [23]. Their activation blocks both oocyte polarisation and karyosome formation. Vasa was proposed to act downstream of the meiotic checkpoint to mediate both oocyte polarisation and karyosome formation [17], [23], but a more recent study suggests that Vasa acts upstream of the checkpoint through involvement in processing of repeat-associated siRNA [24]. Gurken has been shown to be a downstream effector required for oocyte polarisation which is inhibited by the meiotic checkpoint [25], [16], but an effector required for karyosome formation has not been identified.

The karyosome is a compact cluster of meiotic chromosomes formed within the *Drosophila* oocyte nucleus [26] and similar structures are also found in human oocytes [27]. In addition to the successful completion of recombination, recent studies by us and others have shown that nucleosomal histone kinase-1 (NHK-1) is essential for karyosome formation [28], [29]. NHK-1 is a Histone 2A kinase conserved from nematodes to humans (Vrk-1 in *C. elegans*, and Vrk1-3 in mammals) [30]. We showed that NHK-1 also phosphorylates Barrier-to-Autointegration Factor (BAF) to release meiotic chromosomes from the oocyte nuclear envelope during karyosome formation [31]. However nothing is known about how NHK-1 activity itself might be controlled during meiosis.

In this report, we have investigated the functional relationship between NHK-1 and the meiotic checkpoint. We found that the meiotic checkpoint suppresses NHK-1 activity to prevent reorganisation of the oocyte nucleus, including karyosome formation, synaptonemal complex disassembly and condensin loading, until DNA breaks are repaired. Therefore, we propose that NHK-1 is a critical meiotic regulator controlled by the meiotic checkpoint.

## Results/Discussion

### The meiotic checkpoint pathway is not activated in an *nhk-1* mutant

**Figure 1:**
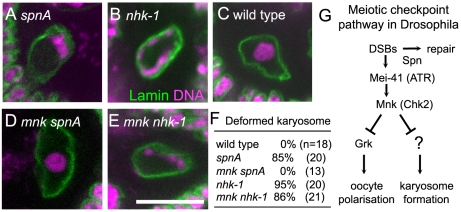
Inactivation of the meiotic checkpoint did not suppress *nhk-1* karyosome defects. The karyosome morphology in an oocyte from *spnA^1^* (A), *nhk-1^E24/Df^* (B), wild type (C), an *mnk^p6^ spnA^1^* double mutant (D), and an *mnk^p6^ nhk-1^E24/Df^* double mutant (E). Bar = 10 µm. (F) The frequency of deformed karyosomes in oocytes with various genotypes. Inactivation of the meiotic checkpoint by the *mnk^p6^* mutation rescued the karyosome defect in *spnA^1^* (p<0.01), but not in *nhk-1^E24/Df^* (G) The meiotic recombination checkpoint pathway in *Drosophila* oocytes (modified from 23).

In the wild-type oocyte nucleus, meiotic chromosomes are clustered together to form a spherical body called the karyosome [26] (Figure 1C). Female sterile *nhk-1* mutations show an abnormal morphology of the karyosome, which is less compact and often attached to the nuclear envelope [28], [29], [31] (Figure 1B and Figure S1). A similar karyosome abnormality is also observed in the *spn* class of mutants, which were originally identified based on their abnormal oocyte polarity [13], [14], [16], [17] (Figure 1A and Figure S1). Most *spn* mutants contain persistent DNA double stranded breaks (DSBs) in meiotic chromosomes and activate the meiotic checkpoint pathway [18]–[22]. Both the karyosome and polarity defects in these *spn* mutants can be rescued by inactivation of the meiotic checkpoint [17], [21], [23] (Figure 1D).

A possible explanation for this similarity in the karyosome defects between *nhk-1* and *spn* mutants is that *nhk-1* mutations lead to an activation of the meiotic checkpoint pathway. To test this possibility, we assessed the activation of the meiotic checkpoint pathway in an *nhk-1* mutant by examining for the persistence of DSBs on meiotic chromosomes and the presence of oocyte/embryo polarity defects. The *nhk-1^Z3-0437^* mutant has been previously shown to have no delay in DSB repair or polarity defects [28]. However, as this allele contains a mis-sense mutation in a residue with unknown function in the kinase domain, the phenotype may be due to the specific nature of this allele. To exclude this possibility, we examined another female sterile allele, *nhk-1^E24/Df^*, that expresses a reduced amount of wild-type NHK-1 protein and shows karyosome defects [29], [31]. To assess oocyte polarity, we examined the dorsal appendages of eggs laid by females, whose formation depends on correct dorsal-ventral axis specification in the oocyte [32]. Dorsal appendages of eggs laid by the *nhk-1^E24/Df^* mutant did not show abnormalities, indicating that oocyte polarity was properly established. Furthermore, immunostaining using an antibody against the phosphorylated form of the *Drosophila* H2AX variant (γ-H2Av) which accumulates at DSB sites [33] showed no detectable DSB foci at late oogenesis stages, indicating DSBs were repaired in the *nhk-1^E24/Df^* mutant (Figure S2). These results indicated that, unlike *spn* mutants, the meiotic checkpoint pathway is not activated in the *nhk-1^E24/Df^* mutant, despite the clear karyosome defect observed in this mutant.

### The karyosome defect in an *nhk-1* mutant does not require meiotic checkpoint activation

To further confirm that the karyosome defect in the *nhk-1^E24/Df^* mutant arises without meiotic checkpoint activation, we examined whether inactivating the checkpoint rescues the karyosome defect in the *nhk-1^E24/Df^* mutant. The meiotic checkpoint signalling pathway contains two kinases, Mei-41 and Mnk, which are homologues of ATM/ATR and Chk-2 respectively (Figure 1G). A mutation in either of these genes has been shown to rescue the karyosome defect caused by unrepaired DSBs in *spn* mutants (Figure 1D) [17], [21], [23], although *mei-41* mutations have been shown to be less proficient at rescuing the karyosome defect probably due to the presence of a second ATM/ATR homologue [21].

We constructed a double mutant between *nhk-1^E24/Df^* and *mnk* by successive genetic crosses, and immunostaining of oocytes was carried out to visualise the oocyte nucleus and the karyosome. This showed that inactivation of the meiotic checkpoint failed to rescue the karyosome defect in the *nhk-1^E24/Df^* mutant (Figure 1E and 1F). In an *mnk nhk-1* double mutant, 86% of oocytes showed deformed karyosome morphology, similar to the *nhk1* single mutant in which 95% of oocytes showed deformed karyosomes (p>0.3). In a control analysis done in parallel, no oocytes from an *mnk spnA* double mutant showed deformed karyosome morphology (Figure 1D and 1F), in comparison to 85% of oocytes from a *spnA* single mutant (p<0.01).

In conclusion, these results demonstrated that the karyosome defect in the *nhk-1^E24/Df^* mutant is not caused by activation of the meiotic checkpoint pathway.

### Unrepaired DSBs suppress NHK-1 kinase activity

The above results demonstrated that the *nhk-1^E24/Df^* mutation induces karyosome defects without activation of the meiotic checkpoint pathway. Therefore, this places NHK-1 function either downstream or in parallel to the meiotic checkpoint pathway. One way to distinguish between these two possibilities would be to examine the kinase activity of NHK-1 in oocytes under conditions activating the meiotic checkpoint pathway (ie. in *spn* mutants). It is known that NHK-1 directly phosphorylates Histone 2A (H2A) at threonine 119 (T119; 30), and this phosphorylation in the oocyte nucleus has been shown to depend on NHK-1 activity [28]. Therefore we decided to examine the level of H2A T119 phosphorylation in the oocyte nucleus as a readout of NHK-1 activity *in vivo* by immunostaining using a phospho-specific antibody (anti-dH2ApT119) [30].

**Figure 2:**
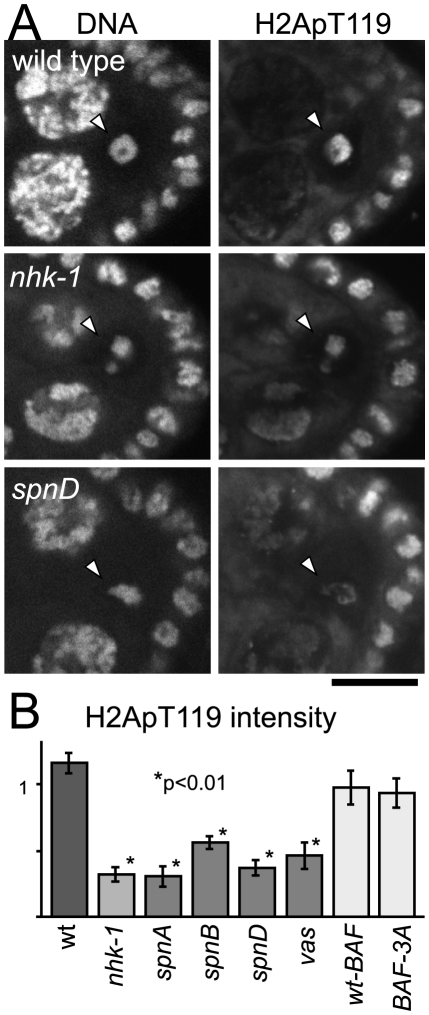
Unrepaired DNA breaks suppress NHK-1 kinase activity. (A) H2A T119 phosphorylation in wild type, *nhk-1^E24/Df^* and *spnD^2^*. Ovaries at stage 5–7 were immunostained with anti-dH2ApT119 antibody and propidium iodide. Arrowheads indicate meiotic chromosomes in oocytes. Bar = 10 µm. (B) The H2ApT119 signal intensity on the chromosomes in oocytes was measured relative to that in follicle cells. The bars on the graph represent standard error of the mean (SEM). A minimum of eight oocytes from each genotype were quantified. NHK-1 activity measured by H2A T119 phosphorylation was significantly reduced in *nhk-1* and *spn* mutant oocytes (p<0.01; marked with asterisks). H2A T119 phosphorylation in oocytes expressing wild-type BAF and non-phosphorylatable BAF (BAF-3A) was comparable to that in wild type, indicating the karyosome abnormality itself is not the cause of low dH2ApT119 signals in *spn* mutants.

Ovaries from *spn* mutants (*spnA*, *spnB*, *spnD* and *vasa*) were dissected and immunostained with the anti-dH2ApT119 antibody. As a control, we also examined wild type and the *nhk-1^E24/Df^* mutant in parallel. Compared to wild type, we found that the H2ApT119 signal was greatly reduced on meiotic chromosomes in oocytes from *spn* mutants, as well as in oocytes from the *nhk-1^E24/Df^* mutant (Figure 2A and Figure S3A).

To quantify the level of the H2ApT119 signal reproducibly and comparably between different oocytes, we measured the H2ApT119 signal in the oocyte nucleus relative to that in follicle cell nuclei, in which H2A T119 phosphorylation has been shown to be independent of NHK-1 activity [28]. H2ApT119 signals in *spn* mutants were significantly reduced (p<0.01; Figure 2B).

We considered the possibility that the reduction of H2ApT119 signal under meiotic checkpoint activation was simply due to abnormal karyosome morphology itself (and we were in fact measuring an artefact or a secondary consequence). First of all, this is unlikely because H2ApT119 signals were also reduced in karyosomes which retained relatively normal morphology in the *spn* mutants (Figure 2A and Figure S3A). To exclude this possibility further, we took advantage of our previous study showing that expressing a non-phosphorylatable version of BAF (a substrate of NHK-1) disrupts the karyosome in these oocytes [31]. Under these conditions, although the karyosome was disrupted, the level of H2ApT119 signal in the oocyte nucleus was comparable to that in oocytes expressing wild-type BAF (that show normal karyosome morphology) or wild-type oocytes (Figure 2B and Figure S3B). Furthermore, to exclude the possibility that the apparent reduction in H2ApT119 was simply due to reduced chromosome condensation or DNA density, we re-quantified H2ApT119 signal intensity relative to DNA staining signal intensity in oocyte nuclei (Figure S3C). The H2ApT119 signal in the oocyte nucleus relative to that in follicle cell nuclei was divided by the DNA staining signal which had been measured using the same method. The result still showed a significant reduction in the H2ApT119 signal relative to DNA signal in *spn* mutant oocytes. The possibility that a simple reduction in H2A levels or its occupancy on DNA accounted for the decrease in H2Ap119 signal was further excluded by immunostaining using a phospho-independent antibody against H2A which did not show reduction in H2A signal in *spn* mutant oocytes (Figure S3D, S3E). These results confirm the genuine suppression of H2A T119 phosphorylation (which infers the suppression of NHK-1 activity) in these mutants.

Therefore we conclude that, judged by the phosphorylation level of one of its substrates, unrepaired DSBs in *spn* mutants suppress NHK-1 kinase activity in the oocyte nucleus.

### The meiotic checkpoint mediates suppression of NHK-1 activity

**Figure 3:**
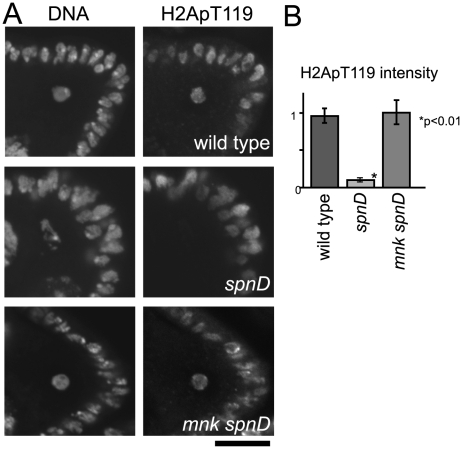
The meiotic recombination checkpoint suppresses NHK-1 activity. (A) H2A T119 phosphorylation in oocytes of wild type, *spnD*, and *mnk spnD*. Mnk (the Chk2 orthologue) is an essential kinase in the meiotic checkpoint. Ovaries at stage 5–7 were immunostained with anti-dH2ApT119 antibody and DAPI. (B) The H2ApT119 signal intensity on the chromosomes in oocytes was measured relative to that in follicle cells. The bars on the graph represent standard error of the mean (SEM). At least ten oocytes from each genotype were quantified. These samples were processed in parallel and compared only with each other, as exact values vary over time due to changes in factors including the conditions of the antibodies and fixative. NHK-1 activity measured by H2A T119 phosphorylation was significantly reduced in oocytes of a *spnD* mutant (p<0.01; marked with an asterisk), but not in those of an *mnk spnD* double mutant. Inactivation of the meiotic checkpoint rescued the suppression of NHK-1 activity in the presence of DSBs.

To confirm whether this suppression of NHK-1 activity by unrepaired DSBs is mediated by the meiotic checkpoint, we tested whether inactivation of the checkpoint (as shown in Figure 1) could abolish this suppression. Inactivation of the checkpoint was achieved by introduction of a mutation in *mnk*, which encodes the crucial checkpoint kinase Chk2. Examination of double mutants between *mnk* and *spnA* and between *mnk* and *spnD* showed that the H2ApT119 signal on meiotic chromosomes in *spn* mutants is restored by inactivation of the checkpoint (Figure 3 and Figure S4). This confirmed that the suppression of NHK-1 activity in the presence of DSBs is mediated by the meiotic checkpoint.

### DNA breaks suppress NHK-1 kinase activity in *Drosophila* cultured cells

Our cytological study showed that DSBs suppress the kinase activity of NHK-1, judged by phosphorylation of its substrate H2A at T119. We wished to confirm this suppression of NHK-1 activity by biochemical means. As biochemical measurements of oocyte-specific NHK-1 activity is challenging, we wondered whether similar suppression of NHK-1 may be observed when DSBs are induced in *Drosophila* cultured cells, without involvement of meiosis-specific factors.

To aid purification of NHK-1 from cultured cells (S2 cells), the NHK-1 gene was fused to GFP in frame and placed under the control of the metallothionein promotor. After transfection into S2 cells, a stable cell line inducibly expressing NHK-1-GFP was established. These cells were irradiated with X-rays at 1 Gy/min for 5 minutes. Immunostaining using a γ-H2Av antibody confirmed that this dose of X-rays efficiently induced DSBs without damaging the ability of cells to repair DSBs (data not shown). Fifteen minutes after X-ray irradiation, cells were collected and NHK-1-GFP was immunoprecipitated from cell extract using a GFP antibody in the presence of phosphatase inhibitors. The kinase activity of immunoprecipitated NHK-1-GFP was assayed *in vitro* by adding radioactive ATP without inclusion of exogenous substrates, as the NHK-1 substrate BAF is co-immunoprecipitated with NHK-1 [31].

**Figure 4:**
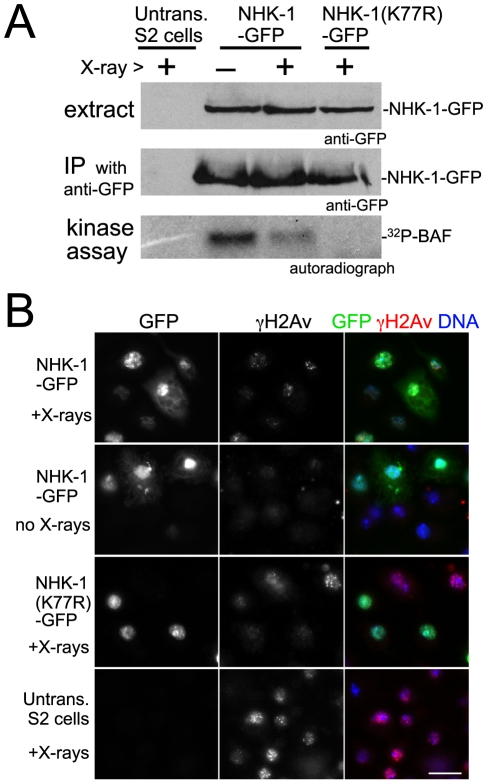
DSBs suppress NHK-1 activity in S2 cells. (A) Kinase activity of NHK-1-GFP was reduced after X-ray irradiation. S2 cells stably expressing NHK-1-GFP or NHK-1(K77R)-GFP, together with untransfected S2 cells, were irradiated with X-rays. Cells were collected 15 minutes later and NHK-1-GFP was immunoprecipitated from cell extracts by a GFP antibody. For the kinase assays, ^32^P-γATP was added and phosphorylation of co-immunoprecipitated BAF by NHK-1 was detected by autoradiograph. Cell extracts and immunoprecipitates used for kinase assays were immunoblotted with a GFP antibody. (B) DSBs were retained and the nuclear localisation of NHK-1-GFP was unaffected when cells were collected after X-ray treatment. DSBs and NHK-1-GFP were detected by immunostaining using antibodies against γH2Av and GFP, respectively. Bar = 10 µm.

Interestingly, we found that *in vitro* phosphorylation of co-immunoprecipitated BAF was greatly reduced in irradiated cells in comparison to non-irradiated cells processed in parallel (Figure 4A). This phosphorylation was dependent on NHK-1 kinase activity, as it was abolished by a mutation in NHK-1 [31] that eliminates its kinase activity but does not interfere with its binding to BAF (Figure 4A). Immunoblotting confirmed that comparable amounts of NHK-1 were immunoprecipitated from irradiated and non-irradiated samples (Figure 4A). When cells were collected 15 minutes after irradiation, their mitotic indexes were comparable (1.2% irradiated vs 1.4% non-irradiated), their nuclei still had unrepaired DSBs and nuclear localisation of NHK-1 was unaffected (Figure 4B). This indicates that the reduction in NHK-1 kinase activity in irradiated cells was not due to a reduction of mitotic cells or a change in NHK-1 localisation.

The suppression of NHK-1 kinase activity after X-ray irradiation was observed in three independent experiments. These biochemical results in S2 cells give further support to our observation in oocytes that NHK-1 kinase activity is suppressed in response to DSBs.

### Activation of the meiotic checkpoint delays other NHK-1 dependent events

In addition to karyosome formation, NHK-1 has been shown to be required for the disassembly of the synaptonemal complex and loading of the condensin complex onto chromosomes during meiosis [28]. Our results showed that the meiotic checkpoint suppresses NHK-1 activity when DSBs are not repaired. From these observations, a prediction is that these other NHK-1 dependent events would also be blocked or delayed when the meiotic checkpoint pathway is activated. Indeed, a previous report showed that disassembly of the synaptonemal complex is delayed in a *spnA* mutant [18].

**Figure 5:**
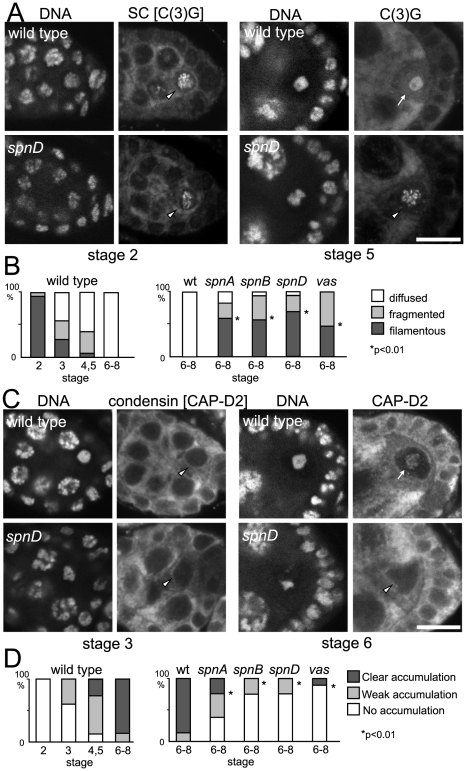
Disassembly of the synaptonemal complex and loading of the condensin complex is delayed by meiotic checkpoint activation. (A) Synaptonemal complex in wild-type and *spn* mutant oocytes. Ovaries were immunostained for the tranverse filament protein C(3)G and DNA. Bar = 10 µm. (B) The C(3)G staining pattern was classified as filamentous (arrowheads in A), fragmented or diffused (arrow in A). *spn* mutants significantly delayed disassembly of the synaptonemal complex (p<0.01; marked with asterisks). A minimum of thirteen oocytes were counted. (C) Condensin in wild-type and *spn* mutant oocytes. Ovaries were immunostained for the condensin subunit CAP-D2 and DNA. Bar = 10 µm. (D) Chromosome accumulation of CAP-D2 staining was classified into clear (arrow in C), weak or absent (arrowheads in C). *spn* mutants significantly delayed condensin loading (p<0.01; marked with asterisks). A minimum of nine oocytes were counted.

To test how universal this is, we examined the disassembly of the synaptonemal complex during oogenesis in various *spn* mutants. Immunostaining using an antibody against the synaptonemal complex protein C(3)G [34] showed that synaptonemal complex disassembly was significantly delayed in *spn* mutants. In wild-type oocytes, synaptonemal complex disassembly was completed by oogenesis stage 6. However, the characteristic filamentous structure of the synaptonemal complex or its remnants were still detected by the C(3)G antibody on meiotic chromosomes even at stage 6 or later in most oocytes of *spn* mutants (*spnA*, *spnB*, *spnD*; Figure 5A and Figure 4B). This delay in synaptonemal complex disassembly in *spn* mutants, but not in the *nhk-1^Z3-0437/Df^* mutant, was reversed by inactivation of the meiotic checkpoint using an *mnk* mutation (Figure S5).

Next we examined condensin loading onto meiotic chromosomes in wild type and *spn* mutants by immunostaining. In wild-type oocytes, the conserved condensin subunit CAP-D2 [35] is fully recruited onto meiotic chromosomes by stage 6 of oogenesis. In *spn* mutants, the protein had not fully accumulated onto meiotic chromosomes in most oocytes even at stage 6 or later (Figure 5C and 5D), indicating that the condensin complex was not fully loaded onto meiotic chromosomes.

These results showed that unrepaired DSBs not only disrupt karyosome formation but also other NHK-1 dependent events. This suggests that suppression of NHK-1 activity plays wider roles in delaying meiotic progression in response to DSBs.

### The meiotic checkpoint suppresses NHK-1 activity to delay nuclear reorganisation in meiosis

These NHK-1 dependent events, disassembly of the synaptonemal complex, loading of condensin and karyosome formation, represent an important transition in oocyte nuclear organisation during meiosis. Temporally karyosome formation takes place at the transition between oogenesis stage 2 and 3 [26] and in fact our quantitative study of synaptonemal complex disassembly and condensin loading in wild-type meiosis (Figure 5B and 5D) indicated that the initiation of these other two NHK-1 dependent events also occurs between oogenesis stage 2 and 3. As these events depend on the presence of a functioning NHK-1 kinase [28], it suggests that NHK-1 is a key meiotic regulator of this important transition in nuclear organisation in oocytes.

It has long been known that activation of the meiotic recombination checkpoint disrupts karyosome formation, and additionally we show here that synaptonemal complex disassembly and condensin loading are delayed by the presence of unrepaired DSBs and an activated checkpoint. It has previously been shown that the meiotic checkpoint blocks oocyte polarisation by suppressing Gurken translation or localisation [16], [17], [23], but it was not previously known how the checkpoint affects karyosome formation or any other nuclear events in oocytes. Our study has shown that the meiotic checkpoint suppresses phosphorylation of an NHK-1 substrate, H2A, when DSBs are not repaired. Furthermore, we found that DSBs induced by X-rays suppress the kinase activity of NHK-1 in S2 cells. These results indicate that NHK-1 is a downstream effector of the meiotic recombination checkpoint, whose suppression is responsible for blocking karyosome formation and other meiotic events until DSBs are repaired.

**Figure 6:**
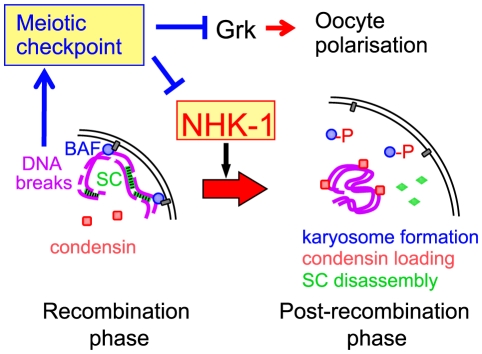
A central role for NHK-1 kinase in meiotic progression. NHK-1 promotes nuclear reorganisation from a recombination phase to a post-recombination phase, including karyosome formation, synaptonemal complex disassembly and condensin loading. NHK-1 directly phosphorylates BAF that anchors meiotic chromosomes to the nuclear envelope. DSBs activate the meiotic recombination checkpoint that suppresses NHK-1 kinase to prevent nuclear reorganisation.

Based on this evidence, we propose a model in which DSBs formed during recombination suppress the activity of the conserved kinase NHK-1 through the meiotic recombination checkpoint to delay oocyte nuclear reorganisation from a recombination to a post-recombination phase (Figure 6). Although the evidence is mostly genetic or cytological, all data are so far consistent with this model. Nevertheless, the model is likely to be too simplistic and to represent only a part of the whole picture. For example, we do not exclude the possibility that other checkpoint effectors are also involved in delaying meiotic progression. We hope that our proposed model will prompt further investigation to fully uncover how the meiotic checkpoint is linked to meiotic progression.

How does NHK-1 kinase control this critical transition in meiosis? Our previous study showed that NHK-1 directly controls karyosome formation through phosphorylation of BAF, a linker between the nuclear envelope and chromatin [31]. Phosphorylation of BAF by NHK-1 releases meiotic chromosomes from tethering at the nuclear envelope to allow karyosome formation. Expression of non-phosphorylatable BAF disrupts karyosome formation, but not synaptonemal complex disassembly or condensin loading (Figure S6). Therefore, NHK-1 appears to control two independent pathways during nuclear reorganisation. This is consistent with a recent study [36] showing that condensin is required for synaptonemal complex disassembly but not for karyosome formation. Karyosome formation and condensin loading are therefore likely to be two primary targets of NHK-1 activity.

Finally, this study in *Drosophila* is likely to have significant implications for our understanding of meiotic regulation at a molecular level in other organisms, since the processes we studied here are conserved among eukaryotes. The meiotic checkpoint that coordinates recombination events with meiotic progression is universally found across eukaryotes. Furthermore, NHK-1 is well conserved among animals, and karyosome-like clustering of meiotic chromosomes, as well as synaptonemal complex disassembly and condensin loading, is widely found in oocytes of various species including humans. In addition, this study has also suggested an involvement of NHK-1 in the DNA damage response during the mitotic cell cycle.

## Materials and Methods

### *Drosophila* genetics

Standard techniques of fly manipulation were followed [37]. All stocks were grown at 25°C on standard cornmeal media except in some case where females were matured at 18°C. General details of mutations, chromosome aberrations and common vectors can be found in [38] or at Flybase [39]. *w^1118^* was used as wild type. The following mutant alleles were used in this study: *nhk-1^E24^* and *nhk-1^Z3-0437^* analysed as a hemizygote over the deficiency *Df(3R)ro-80b* [29], [28]; *spnA^1^* [13], *spnB^1^* [13] analysed as a hemizygote over the deficiency *Df(3R)red3I* and *spnD^2^* [13] that were obtained from the Bloomington Drosophila Stock Centre; *vas^4^* [40]; *mnk^p6^* [21]. Flies containing both *spnA^1^* and *mnk^p6^* mutations were obtained by standard successive genetic crosses. BAF and non-phosphorylatable BAF-3A were expressed from pUASp-BAF and pUASp-BAF-3A transgenes using a maternal Gal4 driver (V2H) under the α-tubulin67C promotor, as previously described [31].

### Immunological and cytological techniques

Standard immunological techniques were used throughout [41]. *Drosophila* ovaries were immunostained essentially as described [42]. Briefly, ovaries were dissected from mature females in Robb's medium (100 mM HEPES, pH7.4, 55 mM sodium acetate, 40 mM potassium acetate, 100 mM sucrose, 10 mM glucose, 1.2 mM MgCl_2_, 1 mM CaCl_2_) before being fixed in formaldehyde (8% paraformaldehyde, 100 mM potassium cacodylate, pH7.2, 100 mM sucrose, 40 mM potassium acetate, 10 mM sodium acetate, 10 mM EGTA). Following a blocking and permeabilization step (in PBS containing 10% foetal bovine serum and 1% Triton X-100), ovaries were successively incubated in primary and secondary antibody solutions before mounting on coverslips in mounting medium (85% glycerol, 2.5% propyl gallate). The primary antibodies used in this study were those against H2ApT119 [30] (1/200), γ-H2Av (this study; 1/100), H2A (Ab13923, Abcam; 1/250), Lamin [43] (1/250), C(3)G [44] (1/3000) and CAP-D2 [35] (1/5000). The antibody against γ-H2Av was generated by Eurogentec using a phospho-peptide (CQRKGNVILpSQAY-COOH), and differentially purified using the phospho-peptide and an equivalent non-phospho-peptide. The antibody gave punctate staining in oocyte nuclei during early oogenesis and in X-ray irradiated nuclei in S2 cells, but not during late oogenesis or non-irradiated S2 nuclei. Secondary antibodies conjugated with Cy3 or Alexa488 (Jackson Lab or Molecular Probes) were used at a 1/100 dilution. DNA was counterstained with DAPI (0.4 µg/ml; Sigma) or propidium iodide (2 µg/ml, Sigma). A series of 1 µm optical sections were taken using a Plan-Apochromat lens (63X, 1.4NA; Zeiss) attached to an Axiovert 200M (Zeiss) with a confocal scan head (LSM510meta; Zeiss) or to an Axioimager (Zeiss) with an LSM5 Exciter (Zeiss). A single mid-section of the oocyte nucleus has been presented. All digital images were imported to Photoshop (Adobe) and adjusted for brightness and contrast uniformly across entire fields.

### Expression of NHK-1-GFP in S2 cells and *in vitro* kinase assay

The culture and transfection of S2 cells were performed as previously described [45]. NHK-1-GFP under the metallothionein promoter was generated by a Gateway-based method (Invitrogen) and NHK-1(K77R)-GFP was made by site-directed mutagenesis. Stable cell lines were created through selection by inclusion of blasticidin in the culture medium. Untransfected S2 cells were used as a control. NHK1-GFP expression from the metallothionein promoter was induced by culturing in medium containing 0.7 mM copper sulfate for 72 h. Aliquotes of cells were adhered to coverlips coated with Concanavalin A for immunostaining for γ-H2Av and GFP. Cells, together with adhered cells, were irradiated with X-rays (1 Gy/minute) for 5 minutes. Subsequently, 2.5×10^7^ cells were lysed in 500 µl of buffer (20 mM Tris pH 7.5, 150 mM NaCl, 5 mM EGTA, 1 mM DTT, 1 mM PMSF, Complete EDTA-free protease inhibitor cocktail (Roche)) supplemented with 10× Protein Phosphatase Inhibitor Cocktail 2 (Sigma).

The cleared lysate was then incubated with 5µl of mouse-anti-GFP antibody (3E6, Molecular Probes) for 1h at 4°C before the addition of 50 µl of 1∶1 protein G beads (Invitrogen) in lysis buffer for 1h at 4°C. The beads were washed with the lysis buffer and kinase reaction buffer (10 mM HEPES pH 7.6, 50 mM KCl, 5 mM MgCl_2_, Complete EDTA-free protease inhibitor cocktail (Roche)) supplemented with 10× Protein Phosphatase Inhibitor Cocktail 2 (Sigma).

In a typical kinase reaction, the suspension of beads and kinase buffer was mixed with 5 µCi of γ-[^32^P]ATP (EasyTides, Perkin Elmer) and incubated at room temperature (20°C) for 60 min before the addition of 20 µl of 2× protein sample buffer. The samples were analyzed by SDS-PAGE, and dried gels were exposed to x-ray films (high performance autoradiography films, GE Healthcare).

### Karyosome image analysis

Analysis of karyosome morphology was performed for images of oocytes stained for lamin and DNA. For each series of images through an oocyte nucleus, the optical sections within which the karyosome was visible were determined. Of these, the mid-optical section was selected for analysis (or the lower of the middle two optical sections where this was the case), and the karyosome morphology categorised. Relative intensities of H2ApT119 or H2A signal on the karyosome in the oocyte nucleus were calculated using images of oocytes stained for H2ApT119 or H2A and DNA. As described above, the mid-optical section within which the karyosome was visible was selected for analysis. Using ImageJ software (NIH), the area corresponding to the karyosome was selected and the maximum H2ApT119 signal intensity on the karyosome was obtained and divided by that in surrounding follicle cell nuclei (an average of maximum signal intensity measurements in three nuclei at similar focal planes) after both intensities had been normalized by subtracting the background maximum signal intensity for a randomly selected area in the oocyte cytoplasm. Relative intensities of DNA staining signal on the karyosome were obtained using the same analysis method, and a measurement of H2ApT119 or H2A signal relative to DNA staining signal on the karyosome was made by dividing the two values for each image. We always compare samples processed in parallel or within a short time frame, as the exact values can vary over time due to a change in various factors including the conditions of the fixative and antibodies. Karyosome staining patterns for oocytes stained for C(3)G or CAP-D2 and counterstained for DNA were categorised as described in ‘Results and Discussion’ and Figure 5. A student t test or χ^2^ test was used for statistical analysis.

## Supporting Information

Figure S1 Variation of karyosome defects in *nhk-1*, *spnA*, *spnB*, *spnD*, and *vas* mutants. (A) Three examples of karyosomes are shown for each mutant. Karyosomes in each mutant are always partially if not extensively localised at the edge of the oocyte nucleus. Similar karyosome morphology phenotypes are observable in *nhk-1* and all *spn* mutants, although the penetrance of the more severe phenotypes is variable between each mutant. Bar = 10 µm. (B) Karyosome morphology was categorised into 5 classes (from the left; spherical and detached from the nuclear membrane (NE), spherical and attached to the NE, deformed and attached to the NE, severely deformed and attached to the NE, deformed and detached from the NE).
(4.31 MB EPS)

Figure S2 Delayed DSB repair was not detected in *nhk-1^E24/Df^* mutant oocytes. Oocytes at stage 5–7 from wild type, *spnA* and *nhk-1^E24/Df^* were immunostained for DNA and phospho-H2Av (γ-H2Av; which accumulates at DSB sites). No phospho-H2Av foci were observed in wild type indicating that DSBs are already repaired. Such foci were observed in a *spnA* mutant due to a failure of DSB repair during recombination. In the *nhk-1* mutant, no phospho-H2Av foci were observed indicating that DSBs were repaired. At least 6 karyosomes were examined. Bar = 10 µm.
(1.44 MB EPS)

Figure S3 Suppression of H2A T119 phosphorylation by DSBs was not due to general karyosome abnormality or a reduction of H2A on chromosomes. (A) H2A T119 phosphorylation in wild-type, *spnA^1^* and *spnB^1^ oocytes*. (B) H2A T119 phosphorylation in wild-type oocytes expressing wild-type BAF (wtBAF) and non-phosphorylatable BAF (BAF-3A) [31]. Ovaries at stage 5-7 were immunostained for H2ApT119 and DNA. Arrowheads indicate meiotic chromosomes in oocytes. Bar = 10 µm. (C) Ratios of signal intensities between phospho-H2A (H2ApT119) and DNA staining in oocytes are shown with standard errors of the mean (SEM). The same oocytes used in Figure 2B were re-analysed. NHK-1 activity measured by H2A T119 phosphorylation was significantly reduced in *nhk-1* and *spn* mutant oocytes (p<0.01; marked with asterisks). (D) Ovaries at stage 5–7 were prepared from wild type, *spnA* and *spnD* as above except a phospho-independent anti-H2A antibody was used. (E) Ratios between H2A and DNA signals in oocytes were quantified as above. No significant differences were observed (p>0.3).
(6.24 MB EPS)

Figure S4 Unrepaired DSBs suppress NHK-1 activity through the meiotic checkpoint. (A) H2A T119 phosphorylation in oocytes of wild type, *spnA* and *mnk spnA*. Ovaries at stage 5–7 were immunostained for H2ApT119 and DNA. Bar = 10 µm. (B) The H2ApT119 signal intensity on the chromosomes in oocytes was measured relative to that in follicle cells. The bars on the graph represent standard error of the mean (SEM). At least ten oocytes from each genotype were quantified. These samples were processed in parallel and compared only with each other, as exact values vary over time due to changes in factors including the conditions of the antibodies and fixative. NHK-1 activity measured by H2A T119 phosphorylation was significantly reduced in oocytes of a *spnA* mutant (p<0.01; marked with an asterisk), but not in those of an *mnk spnA* double mutant. Inactivation of the meiotic checkpoint rescued the suppression of NHK-1 activity in the presence of DSBs.
(5.91 MB EPS)

Figure S5 The delay in synaptonemal complex disassembly in *spn* mutants was mediated by the meiotic recombination checkpoint. (A) Stage 6–8 ovaries from each genotype were immunostained for the synaptonemal complex protein C(3)G and DNA. Bar = 10 µm. (B) The C(3)G staining pattern at stage 6–8 was classified. The delay in synaptonemal complex disassembly in *spn* mutants was reversed by an *mnk* mutation that inactivates the meiotic checkpoint. A minimum of thirteen oocytes were counted. (C) DNA (left) and C(3)G (right) stainings in *nhk-1^Z3-0437/Df^*, *mnk^p6^ nhk-1^Z3-0437/Df^* and wild-type oocytes. Most oocytes in both mutants delay disassembly of filamentous C(3)G structures from chromosomes, and at late stages still have a residual fragmented appearance in the nucleoplasm (included in the “diffused” class; white bars in D). (D) The *mnk* mutation does not rescue the delay in disassembly of the synaptonemal complex in the *nhk-1* mutant. A minimum of ten oocytes were counted.
(4.62 MB EPS)

Figure S6 Expression of non-phosphorylatable BAF does not block condensin loading and synaptonemal complex disassembly. Synaptonemal complex and condensin in wild-type oocytes expressing non-phosphorylatable BAF (BAF-3A) [31]. Ovaries at stage 5–7 were immunostained for the condensin subunit CAP-D2 or the synaptonemal complex protein C(3)G together with Lamin and DNA. Arrowheads indicate fragmented karyosome. Bar = 10 µm.
(1.04 MB EPS)
